# Heat and moisture exchanger use reduces in-hospital complications following total laryngectomy: a case–control study

**DOI:** 10.1186/s40463-016-0154-2

**Published:** 2016-07-07

**Authors:** A. Foreman, R. J. De Santis, F. Sultanov, D. J. Enepekides, K. M. Higgins

**Affiliations:** Department of Otolaryngology Head and Neck Surgery, University of Adelaide, c/o Royal Adelaide Hospital, North Terrace, Adelaide, SA 5000 Australia; Sunnybrook Health Sciences Centre, 2075 Bayview Avenue, Suite M1 102, Toronto, ON M4N 3 M5 Canada; Department of Otolaryngology – Head & Neck Surgery, University of Toronto, Sunnybrook Health Sciences Centre, 2075 Bayview Avenue, Suite M1 102, Toronto, ON M4N 3 M5 Canada

**Keywords:** Heat moisture exchanger, HME, Laryngectomy, Complications, Mucus plugging

## Abstract

**Background:**

Total laryngectomy (TL) is an appropriate oncologic operation for many patients with laryngeal cancer delivering excellent oncologic outcomes, however it remains beset with significant functional consequences. Following TL, the upper and lower airways are permanently disconnected, which causes unfiltered, cold air with reduced humidity to enter the tracheobronchial tree, resulting in mucus overproduction and an increase in the viscosity of the mucus. In response to this, Heat and moisture exchangers were developed to compensate for the lost functions of the upper respiratory tract and their effect on the patients’ respiratory performance in addition to their quality of life.

**Methods:**

The case records of 48 patients undergoing total laryngectomy were reviewed and data concerning demographics, surgical details, post-operative care requirements and adverse events was retrieved. Post hoc analysis of the case patients was undertaken to identify any benefit of using a heat and moisture exchanger (HME) system with particular reference to post-operative respiratory outcomes.

**Results:**

There was no significant difference between case and control subjects based on demographics, extent of surgery or need for flap repair. 16 patients had used a HME and 32 patients had used external humidification (EH). Of those experiencing mucous plugging, only 3/24 (12.5 %) had used a HME system, in contrast to 21/24 (87.5 %) who used EH (Chi square = 9.375, *p* = 0.002). The odds ratio of having an adverse event if not using HME was 8.27 (CI = 1.94 – 35.71). Use of HME also significantly reduced the number of days requiring physiotherapy (1.75 days vs. 3.20 days, *p* = 0.034).

**Conclusion:**

Use of an HME system can reduce in-hospital complications, in particular episodes of mucus plugging, and post-operative care requirements. Furthermore, there is a cost benefit to using HME systems that warrants more widespread introduction of these devices in head and neck surgery centers.

## Background

Total laryngectomy (TL) is an appropriate oncologic operation for many patients with laryngeal cancer delivering excellent oncologic outcomes, however it remains beset with significant functional consequences. Following TL, the upper and lower airways are permanently disconnected and a cervical tracheostoma is created. Whilst this has marked psychosocial consequences for the laryngectomee patient, it also presents a significant physiological challenge to their tracheobronchial tree. The upper airway is responsible for warming, humidifying and filtering the inspired air before it reaches the lower airway. The presence of unfiltered, cold air with reduced humidity causes the tracheobronchial tree mucosa to dry out. This results in mucus overproduction and an increase in the viscosity of the mucus. The combination of these two ultimately lead to mucus plugs and crusts to form, deleteriously affecting mucociliary clearance [1]. Furthermore, tracheobronchial irritation produces metaplasia of the tracheal epithelium. The clinical consequences of this are excessive sputum production, frequent involuntary coughing and repeated forced expectoration to clear the airway, negatively impacting the patient’s quality of life [2].

Heat and moisture exchangers (HME) were introduced almost 30 years ago as a means to compensate for the lost functions of the upper respiratory tract. Additionally, their effect on the patients respiratory performance and their quality of life in the long-term have been well documented [3]. Moreover, it has been noted that it takes at least six weeks to see improvements in pulmonary status and speech outcomes [4, 5]. However even in the post-operative period, the use of HME devices has proven to be effective in improving compliance with humidification delivery, reducing coughing and forced expectoration as well as improving sleeping and patient satisfaction [6]. Nonetheless, the impact of HME devices on significant adverse events in the immediate post-operative period has not previously been reported. We hypothesized that improved compliance with humidification by way of HME usage could reduce the need for escalation of respiratory care and prevent significant adverse events such as the management of mucus plugging. This study aimed to compare total laryngectomy patients who experienced adverse events (mucus plugging) with those who did not, and asses if the proportion of patients who used HME differed between these groups and contributed to improved clinical outcomes.

## Methods

### Study design

The Institutional Review Board of the Sunnybrook Health Sciences Centre approved this study (221–2015). A case–control study of consecutive total laryngectomy patients was undertaken to document cases of post-operative complications of mucus plugging, during the patient’s hospital stay and to identify whether or not the use of HME influenced these occurrences. Analysis on the balance of 1) previous radiation exposure to the larynx and 2) presence of pre-existing respiratory conditions (COPD or Asthma), in the case and control groups, was conducted to ensure that observed results could not be attributed to these potential confounding factors. Subsequently, if a significant difference in HME use existed between these groups, post-hoc analysis will be utilized to identify if patients using HME had improved clinical outcomes in respect to their post-operative care requirements and their respiratory outcome.

### Data acquisition

All Patients who underwent total laryngectomy at Sunnybrook Health Sciences Centre by one of two attending surgeons (KH, DE) were included in the study. Data was collected by a single reviewer (RDS) and patients were assigned to the case group if they experienced post-operative adverse events (mucous plugging). All other patients were placed into the control group. Demographic data (age, gender) and surgical data (extent of pharyngectomy, use of flap closure, type of flap closure) were obtained from the case records. Furthermore, details of the post-operative course including days in ICU, days on the general ward, frequency of suctioning, frequency of stoma care, number of days requiring suctioning and number of days chest physiotherapy was received (either physiotherapist-led or nurse-led) were recorded. Finally, escalation of treatment indicated by increased humidification requirements, was recorded. After patients were placed into their respective groups, the use of, or an absence of an HME device was established; the cost of using HME for each patient was collected.

### Statistical analysis

Data was collected for the entire course of the patient’s post-operative course with the clinical endpoint being hospital discharge. Summary statistics were produced for the clinical and demographic variables. Case and control subjects were matched based on gender initially, then on age (within five years) and finally the need for flap reconstruction of the pharyngeal defect. Gender matching was performed first as there were significantly more males in the study population. Age may reflect physiological performance and the need for free flap reconstruction indicated the extent of the operation. All of these factors were considered significant in predicting adverse outcomes. Matched variables were compared between the case and control groups to ensure the variables were balanced. Examination of the relationship between the case and control groups with the use of an HME was evaluated using a chi square test. Additionally, chi square analysis was used to assess if there was a statistically significant difference in the prevalence of confounding factors (previous radiation to the larynx or pre-existing respiratory conditions) across the case and control groups. Post-hoc analysis, if appropriate, compared patients who used an HME with those who utilized an external humification apparatus (EH). These patient groups were compared on their length of hospital stay, days in ICU, frequency of stoma care, days requiring suctioning, days physiotherapy was conducted, frequency of nurse-led chest physiotherapy, amount of mucus, sleep quality or need for escalation of humidification. For all tests, the difference between the groups was examined via either the chi square test for categorical variables or *t*-test for continuous variables. Where appropriate for small value groups, Fisher’s exact test was used in place of the chi square test. *p* < 0.05 was set as the significance level. All statistics were performed using SPSS V20 (IBM Corp, Armonk, NY).

### Description of Care

All patients who had a free flap were monitored for at least 24 h in the ICU and then transferred to the ward when medically stable. All patients who did not have free flap reconstruction were monitored in a ward-based step-down unit with 2:1 nursing care until medically stable for routine nursing care.

## Results

### Demographic data

48 patients were enrolled in this study during the recruitment period. The study group included 38 males and 10 females. The mean age between groups was not significantly different (case = 65.33 years, SD = 10.89, control = 61.54 years, SD = 11.60, *p* = 0.249). Additionally, the matched variables proved to be balanced between the case and control group with no statistically significant difference observed in the distribution of sex (*p* = 0.724), the extent of a pharyngectomy (*p* = 0.283), or type of flap used in surgery (*p* = 0.124). Sixteen patients used a Provox (Atos Medical Inc, West Allis, WI) HME device in the post-operative period and 32 were managed with EH.

### Surgical details

26 patients had total laryngectomy alone, whereas of the 22 patients who also had pharyngeal resection, 19 had a partial pharyngectomy and 3 had a total laryngopharyngectomy. This was not statistically different between groups (*p* = 0.283). Flap reconstruction was required in 31 patients. The range of flaps used included radial forearm free flap (*n* = 3), anterolateral thigh free flap (*n* = 14), temporoparietal fascial flap (*n* = 11), pectoralis major flap (*n* = 2) and sternocleidomastoid flap (*n* = 1). Again there was no difference between the case and control groups (*p* = 0.124).

### Adverse events

**Fig. 1:**
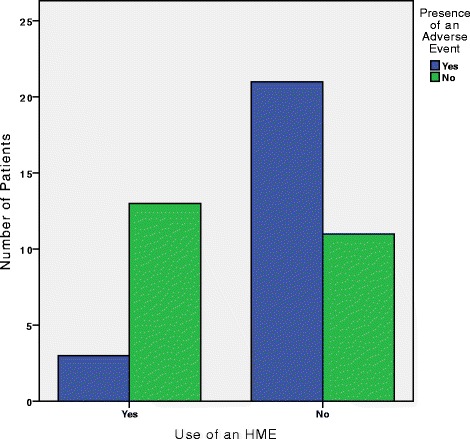
Presence of adverse events based on HME use. Use of HME reduced the chances of an adverse event by an OR of 8.27. Of the 24 patients with an adverse event, 3 used an HME and 21 used EH. However 13 of 24 patients without an adverse event used an HME, and 11 did not

In this study adverse events were defined as mucus plugging. 24/48 (50 %) experienced mucus plugging in the post-operative period. Of those experiencing an adverse event, 3/24 (12.5 %) had used a HME system, in contrast to 21/24 (87.5 %) who used EH (Fig. 1). There was a significant difference between case and control groups based on use of an HME (Chi square = 9.375, *p* = 0.002). The odds ratio (OR) of having an adverse event if not using HME was 8.27 (CI = 1.94 – 35.71). Additionally, chi square analysis revealed that there was no statistically significant difference in prevalence of previous radiation exposure (Table 1) to the larynx between the case (14/24) and control (11/24) groups (Chi square = 0.751, *p* = 0.564). The same conclusion can be made from examining the prevalence of pre-existing respiratory conditions (Table 2) between the case (3/24) and control (4/24) groups (Chi square = 0.167, *p* = 1). More information on the potential confounding variables can be found in Table 1. Since the total laryngectomy patients with adverse outcomes had statistically significantly fewer patients treated with an HME, and there was no statistically significant relationship in the distribution of the above potential confounding variables, post-hoc analysis was warranted. The results from this are reported in the sections below.

**Table 1:** Prevalence of Previous Radiation to the Larynx

	Case	Control
Proportion who received radiation	14 of 24	11 of 24
Average Dosage	46.9 Gy	58.4 Gy
Range	8 Gy–70 Gy	20 Gy–70 Gy

**Table 2:** Prevalence of Pre-existing respiratory conditions

Type of Respiratory Condition	Case	Control
Number of patients with COPD	3	2
Number of Patients with Asthma	0	2

### Post-operative care outcomes

Patients using HME had a non-significant reduction in their length of hospital stay (17.5 days, SD = 12.90 vs. 22.11 days, SD =20.56; *p* = 0.418) and fewer days in the intensive care unit (1.53 days, SD = 1.28 vs. 1.67, SD = 2.76; *p* = 0.848). There was no difference between the daily frequencies of stoma care requirements (2.34 episodes, SD = 0.63 vs. 2.30, SD = 0.51; *p* = 0.781). These post-operative care outcomes are also listed in Table 3.

**Table 3:** Clinical outcomes for HME vs. EH

Variable	Mean HME	Mean EH	*P* Value
Days in Hospital	17.5 (SD = 12.90)	22.11 (SD = 20.56)	0.418
Days in ICU	1.53 (SD = 1.28)	1.67 (SD = 2.79)	0.848
Suctioning Per Day	2.47 (SD = 1.07)	2.30 (SD = 0.94)	0.572
Frequency of Stoma Care (times cleaned per day)	2.34 (SD = 0.63)	2.30 (SD = 0.51)	0.781
Days Requiring Suction	9.81 (SD = 5.05)	10.09 (SD = 8.30)	0.902
Days of PT	1.75 (SD = 0.31)	3.20 (SD = 0.59)	0.034

### Respiratory outcomes

The HME group had a non-significant reduction in cumulative days of suctioning (9.81 days, SD = 5.05 vs. 10.09, SD = 8.30; *p* = 0.902). However, there was a significant reduction in the days requiring chest physiotherapy in the HME user group (1.75 days, SD =1.24 vs. 3.20 days, SD = 0.59; *p* = 0.034). There was no significant difference between groups based on frequency of nurse-led chest physiotherapy, amount of mucus, sleep quality or need for escalation of humidification. These respiratory outcomes are listed in Table 3.

### Cost analysis

The cost of a single HME cassette is C$3.13. The patients used a mean of 28.9 cassettes (SD = 11.77) during their hospital stay producing a mean in-hospital cost of C$90.51 (SD = C$36.85) for the device itself.

## Discussion

Respiratory function following TL is a significant problem for both patients and surgeons that represents an area of ongoing investigation in order to try to optimize outcomes in this set of patients. The physiological insult that results from TL can have a devastating effect on the lower respiratory tract, which is unaccustomed to the cold, dry, and unfiltered air that it is presented with following disconnection of the upper and lower airways. The increase in mucus production and viscosity along with secondary dysfunction of the mucociliary elevator reveals itself clinically as frequent, involuntary coughing, excess sputum production and repeated forced expectoration to try to clear the airways [2, 7]. All of these affect the patient’s quality of life and ability to return to his or hers pre-operative level of functioning following TL. These symptoms typically increase over a period of six to twelve months before stabilizing thereafter. It is important to remember that the majority of patients undergoing TL for laryngeal cancers are current or reformed cigarette smokers and therefore are likely to have a degree of concomitant lower airway disease. In the context of the aforementioned respiratory consequences of TL, it becomes clear that this operation can readily aggravate an already precarious functional state [8].

Humidification following TL has traditionally been provided by way of an external humidification (EH) device where an electrical heater is connected to an air-driven evaporator that directly applies moisturized and warmed air to a mask over the cervical tracheostoma [6]. These systems are noisy, which impacts sleep, and confine the patient to the bed or close by to the EH. The HME device was developed as an alternative to EH that could provide the same, or better, humidification, warming and filtration to the inspired air via a body-worn device. This enables the patient to mobilize normally and also removes the EH unit from their room. The expired air moisturizes the HME filter, which then warms and filters particles during inspiration. An additional benefit is the slight increase in pulmonary resistance, which can provide a positive end expiratory pressure to splint the distal airways open [2]. This final function does take some adjustment on the part of the patient. Patient compliance has been proven to be essential to the long-term benefits of HME use [8, 9]. The early adoption of HME in the early post-operative period enables the patient to become immediately accustomed to the increased airway resistance the device provides rather than attempting to adapt to this at a later date.

Whilst the long-term efficacy of HME systems has been extensively reported, this study is the first to report a reduction in significant in-hospital adverse events. Whilst much focus of HME research has been in detailing the long-term efficacy, our study highlights the potential benefit of HME systems in minimizing respiratory decompensation early in the post-operative period. Therefore, in addition to the established benefit of helping the patient adjust to the increased airway resistance provided by a HME, we propose that early adoption of HME devices can reduce the incidence of in-hospital adverse events, which has significant implications for the patient, surgeon and hospital.

HME devices were originally designed in Europe and much of the current literature pertains to their use on that continent where they have become an accepted standard of care. The adoption of HME in North America is only relatively recent and we believe, whilst other HME devices have been used, this is the first published series to report the introduction of the Provox HME devices into head and neck surgery practice in Canada [10]. Given the demonstrated short and long-term benefits of the HME, the cost is relatively low. Whilst direct comparison between EH and HME was not possible due to the retrospective nature of the data collection, cost data was collected prospectively for the patients who used an HME device. The mean in-hospital cost of using an HME was $90.51 (SD = $36.85) for the device itself. The patients using the device had a range of 9–21 days spent in the hospital, each using 1–3 HME cassettes a day. Thus, overall, the total amount of HME cassettes used ranged from 16–47. The fact that each patient is unique, and thus requires a unique care plan, resulted in the variation in the amount of HME devices needed. This explains the large standard deviation reported for the average price of using the HME device. Moreover, cost savings are made in terms of number of disposable suction catheters making introduction of HME almost cost neutral. The ultimate saving is in patient overall morbidity following TL.

## Conclusion

HME has well-established long-term efficacy in improving respiratory outcomes following TL. In the first reported series from Canada, we have demonstrated that there is a significant reduction in in-hospital complications with HME use following TL. Coupled with its well known long-term benefits the current data supports the more widespread introduction of these devices in head and neck surgical practice across Canada and the rest of North America.

## Abbreviations

EH, External Humidification; HME- Heat and Moisture Exchanger; TL, Total Laryngectomy
